# Complete Proximal Right Coronary Artery Occlusion in a Patient With Normal Initial Acute Coronary Syndrome (ACS) Workup: The Diagnostic Value of Clinical Judgment and Risk Stratification

**DOI:** 10.7759/cureus.98139

**Published:** 2025-11-30

**Authors:** Taner B Celebi, Alvin Stanley, Supriya Baskaran, Nicole Volino, Steven Savella

**Affiliations:** 1 Family Medicine, Northwell Health, Commack, USA; 2 Cardiology, New York Institute of Technology College of Osteopathic Medicine, Old Westbury, USA; 3 Family Medicine, Northwell Health, Plainview, USA; 4 Cardiology, Northwell Health, Manhasset, USA

**Keywords:** acute coronary syndrome (acs) and stemi, cardiac computed tomography angiography, cardiac troponin, coronary artery angiography, coronary artery disease (cad), coronary stents, medicine history, stable angina, timi score, unstable angina

## Abstract

Acute coronary syndromes (ACS), including unstable angina, non-ST-elevation myocardial infarction (NSTEMI), and ST-elevation myocardial infarction (STEMI), are major causes of emergency department (ED) visits and are typically evaluated using clinical presentation, electrocardiogram (EKG), and cardiac biomarkers such as troponin. We present the case of a 47-year-old male with hyperlipidemia and a coronary artery calcium score (CACS) >200 who reported worsening exertional chest pain and dyspnea. Despite a normal EKG, serial troponins, prior stress test, and echocardiogram, his symptoms raised clinical concern. He was admitted with suspected unstable angina, and cardiac catheterization revealed a 100% occlusion of the proximal right coronary artery (pRCA), successfully treated with a drug-eluting stent. His symptoms resolved completely within three months. This case highlights the limitations of relying solely on non-invasive testing and biomarkers in detecting significant coronary artery disease (CAD), especially when collateral circulation is present. It also emphasizes the value of clinical judgment and the integration of risk stratification tools such as the Thrombolysis in Myocardial Infarction (TIMI) and​​​​​​​ History, Electrocardiogram, Age, Risk factors, and Troponin (HEART) scores. Ultimately, this case underscores the need for a vigilant, individualized approach to chest pain evaluation, where early invasive diagnostics may be warranted even in the absence of definitive initial findings.

## Introduction

Acute coronary syndromes (ACS) are a group of conditions resulting from a sudden reduction of blood flow to the heart, typically as a result of atherosclerosis of the coronary vessels [1]. This group is composed of unstable angina, non-ST-elevation myocardial infarction (NSTEMI), and ST-elevation myocardial infarction (STEMI). These syndromes all involve cardiac ischemia, but can be distinguished based on clinical presentation, EKG findings, and cardiac biomarkers. The least dangerous of the ACS is unstable angina. The two most common causes of unstable angina are due to nonocclusive thrombus formation on an atherosclerotic plaque or vasospasm of a coronary artery, the latter being less common than Prinzmetal angina. Unstable angina may present as pressure-like chest discomfort with associated difficulty breathing, diaphoresis, nausea, and/or vomiting [1]. The pain may radiate to the shoulders and may improve with rest and worsen with exertion. Pain can occur during both exertion and at rest, making it more serious than stable angina.

EKG will typically show ST-segment depression, T-wave inversions, or appear normal. Troponins will also either be negative or only mildly elevated. This is in contrast with NSTEMIs, which showcase similar symptoms and EKG findings but contrarily have an elevated troponin level. A STEMI, on the other hand, is a medical emergency that is typically associated with EKG changes and elevated cardiac markers such as troponin. Thus, troponin is an important measurement needed for the diagnosis of NSTEMI and STEMI. Cardiac troponin I (cTnI) and T (cTnT) are essential components of the myocardial contractile apparatus and are predominantly expressed in the heart [2,3]. Therefore, elevations in cTnI and cTnT are highly indicative of myocardial injury. Myocardial injury is identified when blood levels of cardiac troponins (cTn) exceed the 99th percentile of the upper reference limit [2]. According to the Fourth Universal Definition of Myocardial Ischemia, detection of a rise and/or fall in cTn values - with at least one value above the 99th percentile URL - together with at least one of the following: symptoms of acute myocardial ischemia, new ischemic EKG changes, newly appearing pathologic Q waves, imaging evidence consistent with ischemic etiology or identification of a coronary thrombus via angiography, must be present in order to diagnose with a type 1 myocardial infarction [2,4].

We present the unique and rare case of a 47-year-old male who presented to the emergency department (ED) with chest pain and other typical cardiac symptoms whose initial diagnostic testing, including serial troponins, electrocardiography (EKG), echocardiography, and stress testing, failed to reveal ischemia. However, cardiac catheterization demonstrated a 100% occlusion of the right coronary artery. This case demonstrates the importance of clinical judgement in the evaluation of cardiac symptoms even in the absence of traditional markers of myocardial infarction.

## Case presentation

This is a 47-year-old male with a past medical history of hyperlipidemia (HLD), gastroesophageal reflux disease (GERD), anxiety and former tobacco smoker of around six pack-years in his 20s, and no familial history of cardiac arrest who is presenting to the ED of a secondary community based center complaining of intermittent exertional substernal chest pain for the last few months that has been progressively worsening in frequency and intensity, especially in the last two weeks. The patient stated that he was not able to walk to his car without stopping multiple times due to shortness of breath and chest tightness. Symptoms would resolve with rest. The patient denied any fever, chills, nausea, vomiting, diaphoresis, leg swelling, or recent travel. Review of systems was otherwise unremarkable. About six months prior, the patient stated he had seen a cardiologist for a routine check-up. At the time, a cardiac stress test and echocardiogram were performed and came back normal, but had an elevated coronary artery calcium score (CACS) that was greater than 200, and was prescribed atorvastatin 20 mg. His other home medications, including clonazepam, citalopram, and pantoprazole, were taken as needed.

**Figure 1:**
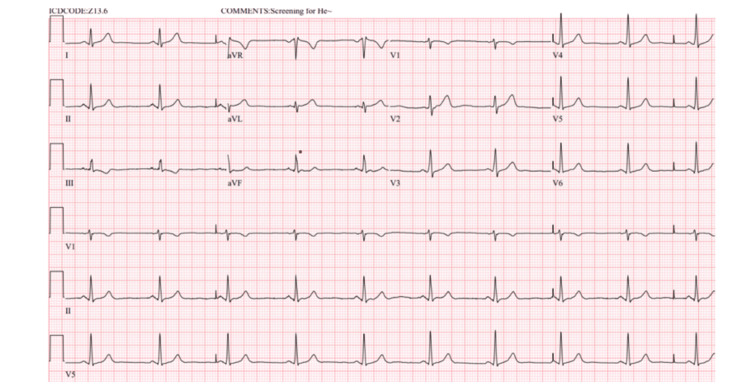
Electrocardiogram obtained on presentation illustrating sinus bradycardia.

Upon presentation in the ED, the patient’s vital signs were within normal limits: temperature 98.0°F (36.7°C), heart rate 62 bpm, blood pressure 124/82 mmHg, respiratory rate 16 breaths per minute, and oxygen saturation 96% on room air. The patient’s weight was 184.9 lbs (83.9 kg) and height was 6 ft. There were no abnormalities upon physical examination, including regular rate and rhythm with normal S1 and S2 heart sounds on cardiac exam, and lungs were clear to auscultation bilaterally on respiratory exam. Comprehensive metabolic panel (CMP) was unremarkable. Complete blood count (CBC) with automated differential revealed a slightly decreased hemoglobin and hematocrit at 12.8 g/dL and 36.7%, respectively, but were otherwise unremarkable. Coagulation studies revealed no abnormalities. Cardiac biomarkers were within normal limits. Specifically, creatine kinase-MB (CK-MB) was <1.0 ng/mL. The first high-sensitivity troponin I measurement was 5.9 ng/L, and three hours later, the second measurement was 6.3 ng/L (Table 1). Chest X-ray was negative for pneumothorax, opacities, or free air. Electrocardiogram (EKG) revealed sinus bradycardia with no acute ST/T wave abnormalities, as seen in Figure 1.

**Table 1:** The patient’s initial laboratory values at presentation.

Lab	Patient's Value	Reference Range
Hemoglobin	12.8	13.0-17.0 g/dL
Hematocrit	36.7	39.0-50.0%
Creatine kinase-MB (CK-MB)	<1.0	0.0-3.6 ng/mL
Initial high-sensitivity troponin	5.9	≤78.5 ng/L
Three-hour high-sensitivity troponin	6.3	≤78.5 ng/L

Despite the lack of abnormalities in pertinent lab findings, the patient was admitted to the telemetry unit by the cardiology team with a primary diagnosis of unstable angina due to a concerning history and was scheduled for cardiac catheterization. The patient was given one aspirin 324 mg oral tablet and took his routine atorvastatin 20 mg oral tablet at bedtime.

The patient was loaded with ticagrelor 180 mg and started on 90 mg twice daily thereafter, along with continuing aspirin 81 mg daily. A transthoracic echocardiogram (TTE) was also performed to evaluate left ventricular ejection fraction (LVEF) and revealed calcification of the mitral valve annulus and mild mitral regurgitation, but otherwise normal functioning heart with a LVEF of 64%.

**Figure 2:**
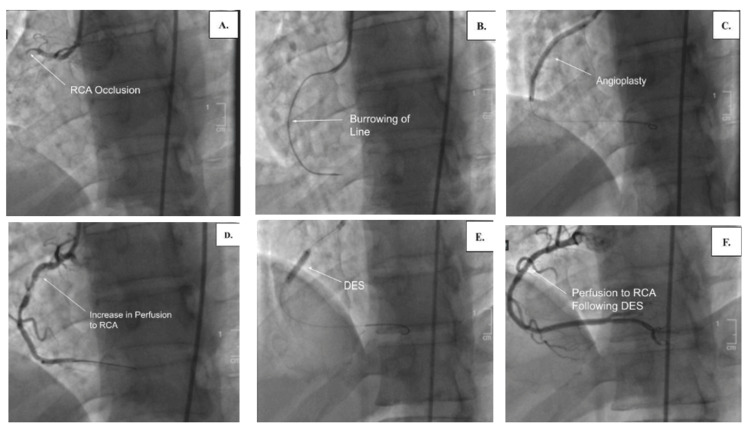
Coronary angiography of the patient. Coronary angiography demonstrating complete occlusion of the proximal right coronary artery (pRCA) (A), advancement through the thrombus (B), balloon angioplasty (C), restoration of perfusion post-angioplasty (D), deployment of a drug-eluting stent (DES) (E), and restored perfusion to the right coronary artery (RCA) after DES placement (F).

The patient was then transferred to another hospital to undergo a percutaneous coronary intervention. Cardiac catheterization was performed the following day and revealed a 100% occlusion of the proximal right coronary artery (pRCA) by an organized thrombus and left-to-right collaterals. Illustration of occlusion, burrowing through the thrombus, angioplasty, perfusion following angioplasty, stenting, and illustration of perfusion of stenting can be seen in Figure 2.

A drug-eluting stent (DES) was placed at the pRCA via right femoral artery (RFA) access. The procedure followed protocol and was uncomplicated. After the procedure, the patient remained hemodynamically stable and neurovascularly intact, with no chest pain and no new EKG changes. He remained overnight for observation and pain control; however, the rest of his hospitalization was uneventful. On day 4, he was provided education regarding dual antiplatelet therapy (DAPT) and medication adherence to his statin upon discharge. The patient was to follow up with his private cardiologist in two weeks and was advised to return to the ER with any concerning symptoms.

Following a three-month follow-up, the patient acknowledged significant improvement in his shortness of breath and his activity level. He was now able to participate in all his previous exercises and extracurricular activities.

## Discussion

The chief complaint of chest pain accounts for nearly 11 million encounters in the ED each year, making up more than 5% of ED visits [5]. Differentiating cardiac causes from non-cardiac causes can be difficult, especially if the patient appears well. Therefore, it is important to rule out cardiac causes immediately through an initial workup. Initial cardiac workup, at a minimum, includes an EKG within the first 10 minutes of arrival, a CBC, a CMP, a chest X-ray, and serial troponin levels [6].

In this patient’s case, his EKG didn’t show any abnormalities that pointed toward ACS. Serial troponins and other biomarkers were also negative. However, this case highlights an unusual presentation of a patient complaining of chest pain with a negative initial cardiac workup, but ended up having a completely occluded pRCA upon cardiac catheterization. Despite the diagnostic challenges, clinical suspicion from the history of the patient’s present illness and presenting risk factors led to the hospital admission of the patient.

Risk stratification in the ED can be done using Thrombolysis in Myocardial Infarction (TIMI) and History, Electrocardiogram, Age, Risk factors, and Troponin (HEART) scores. The TIMI risk score is used to estimate mortality in patients with unstable angina and NSTEMI [7]. The HEART score is used to predict the risk of a major cardiac event occurring in six weeks in a patient presenting with chest pain [8]. Both scores are explained in further detail in Tables 2-3, respectively.

**Table 2:** TIMI risk score. The Thrombolysis in Myocardial Infarction (TIMI) scoring system was used in risk stratification [7].

Age ≥ 65	+1	Calculated Score	Risk %
≥3 Coronary artery disease (CAD) risk factors: Family history of CAD, hypertension, hypercholesterolemia, diabetes, or current smoker	+1	0-1	5%
Known CAD (stenosis ≥50%)	+1	2	8%
Aspirin use in the past seven days	+1	3	13%
Severe angina (≥2 episodes in 24 hrs)	+1	4	20%
EKG ST changes ≥0.5mm	+1	5	26%
Positive cardiac marker	+1	6-7	41%

**Table 3:** HEART score. The HEART (History, ECG, Age, Risk factors, Troponin) scoring system was used for this risk stratification [8]. HTN: hypertension; DM: diabetes mellitus; CVD: cardiovascular disease

	+ 0 points	+ 1 point	+ 2 points	Calculated score	Risk %
History	Slightly suspicious	Moderately suspicious	Highly suspicious	0-3	0.9-1.7%
EKG	Normal	Non-specific repolarization disturbance	Significant ST deviation	4-6	12-16.6%
Age (years)	<45	45-64	≥65		
Risk factors: HTN, hypercholesterolemia, DM, obesity (BMI >30 kg/m^2^), smoking (current, or smoking cessation ≤3 months), positive family history (parent or sibling with CVD before age 65)	No known risk factors	1-2 risk factors	≥3 risk factors or history of atherosclerotic disease	7-10	50-65%
Initial troponin	≤normal limit	1-3× normal limit	>3× normal limit		

Additionally, his cardiac stress test and echocardiogram were normal just six months prior. Both of these are key diagnostic tools used in the evaluation of coronary artery disease (CAD) and the future risk of cardiac complications. A cardiac stress test assesses the heart's function under stress, typically induced by exercise or pharmacologic agents. It is used to diagnose CAD, evaluate the severity of ischemia, and assess prognosis in patients with known or suspected heart disease [9,10]. An echocardiogram uses ultrasound to create images of the heart, providing detailed information about cardiac structure and function, as it can visualize left and right ventricular function, regional wall motion abnormalities, and structural heart diseases [11]. Combining stress testing with echocardiography enhances diagnostic accuracy for CAD. It evaluates myocardial ischemia by detecting stress-induced wall motion abnormalities and provides information beyond clinical history and ECG findings [10]. Thus, the lack of abnormalities on the cardiac stress test and echocardiogram is noteworthy, as these results did not foreshadow what would occur within the next six months. However, this patient’s elevated CACS was concerning and did not correlate with the normal findings of the other tests. CACS is another widely used tool to assess the risk of CAD and helps quantify calcified blockage based on a non-contrast cardiac computed tomography (CT) scan. The score can be interpreted into CAD progression risk, as seen in Table 4 [12]. This patient's CACS was measured to be more than 200, which classifies him as moderately increased risk for cardiovascular events in the future. In turn, the patient was started on a statin at the time. However, such a high CACS should have prompted further testing with tighter follow-up or earlier intervention to have prevented the event.

**Table 4:** CACS interpretation and considerations. CACS: coronary artery calcium score; CAD: coronary artery disease Source: Reference [9]

Score	Interpretation	Considerations
0	Very low risk of future coronary events	No need to start statin medications unless other high-risk factors are present
1-100	Low-significant CAD risk	Consider statin therapy and lifestyle modifications
101-400	Moderately increased significant CAD risk	Strong consideration for statins and closer follow-up
>400	High risk for myocardial infarction	Aggressive prevention and regular monitoring

This patient’s preserved LVEF and normal ECG findings revealed the significance of collateral circulation. A completely occluded coronary artery, as seen in this patient, remained undetected until he presented to the ED with concerning symptoms. The left-to-right collateral vessels maintain sufficient myocardial perfusion, preventing transmural ischemia to the left ventricular wall. Thus, standard non-invasive diagnostic evaluations were not able to reveal underlying CAD in such cases. Based on multiple factors such as genetics, severity of stenosis, and other cardiovascular risk factors, angiogenesis can occur within one to two days [13,14]. However, when the patient began to become increasingly symptomatic, it can be inferred that the collaterals were no longer sufficient for the level of exercise he was doing. The intervention of the cardiac catheterization was ultimately what simultaneously diagnosed and treated the patients' symptoms, with resolutions of symptoms occurring within three months.

## Conclusions

This case underscores the complexity and nuance involved in evaluating chest pain in the ED, particularly when standard initial diagnostics appear reassuring. Despite a normal EKG, serial troponins, and recent non-invasive cardiac testing, including a stress test and echocardiogram, this patient was ultimately found to have a completely occluded pRCA, identified only through cardiac catheterization. His case highlights the importance of clinical judgment and the need for heightened vigilance in the presence of concerning symptoms and risk factors, even in the absence of definitive findings. The elevated CACS obtained six months earlier was an early warning sign of significant underlying CAD, despite normal findings on stress testing. This suggests that CACS should be given more weight in risk stratification and may warrant more aggressive follow-up and diagnostic evaluation. Additionally, the role of collateral circulation masked the severity of his condition until symptom progression outpaced compensatory perfusion.

Ultimately, this case emphasizes that a high index of suspicion, combined with clinical context and individualized risk assessment tools such as the TIMI and HEART scores, remains critical in guiding decision-making. Early intervention through percutaneous coronary intervention resulted in full resolution of symptoms and restoration of functional capacity, reaffirming the value of timely and thorough cardiovascular evaluation in patients with atypical or subacute presentations.

## References

[1] Goyal A, Singh B, Ahmed I (2025). Unstable angina. StatPearls [Internet].

[2] Thygesen K, Alpert JS, Jaffe AS, Chaitman BR, Bax JJ, Morrow DA, White HD (2018). Fourth universal definition of myocardial infarction (2018). Circulation.

[3] Bhatt DL, Lopes RD, Harrington RA (2022). Diagnosis and treatment of acute coronary syndromes: a review. JAMA.

[4] Bergmark BA, Mathenge N, Merlini PA, Lawrence-Wright MB, Giugliano RP (2022). Acute coronary syndromes. Lancet.

[5] Yukselen Z, Majmundar V, Dasari M, Arun Kumar P, Singh Y (2024). Chest pain risk stratification in the emergency department: current perspectives. Open Access Emerg Med.

[6] Johnson K, Ghassemzadeh S (2022). Chest pain. StatPearls [Internet].

[7] Antman EM, Cohen M, Bernink PJ (2000). The TIMI risk score for unstable angina/non-ST elevation MI: a method for prognostication and therapeutic decision making. JAMA.

[8] Six AJ, Backus BE, Kelder JC (2008). Chest pain in the emergency room: value of the HEART score. Neth Heart J.

[9] Agatston AS, Janowitz WR, Hildner FJ, Zusmer NR, Viamonte M Jr, Detrano R (1990). Quantification of coronary artery calcium using ultrafast computed tomography. J Am Coll Cardiol.

[10] Fletcher GF, Ades PA, Kligfield P (2013). Exercise standards for testing and training: a scientific statement from the American Heart Association. Circulation.

[11] Gulati M, Levy PD, Mukherjee D (2021). 2021 AHA/ACC/ASE/CHEST/SAEM/SCCT/SCMR guideline for the evaluation and diagnosis of chest pain: a report of the American College of Cardiology/American Heart Association Joint Committee on Clinical Practice Guidelines. Circulation.

[12] Gać P, Jaworski A, Parfianowicz A, Karwacki J, Wysocki A, Poręba R (2024). Discrepancies between coronary artery calcium score and coronary artery disease severity in computed tomography angiography studies. Diagnostics (Basel).

[13] Stoller M, Seiler C (2015). Salient features of the coronary collateral circulation and its clinical relevance. Swiss Med Wkly.

[14] de Groot D, Pasterkamp G, Hoefer IE (2009). Cardiovascular risk factors and collateral artery formation. Eur J Clin Invest.

